# Medically induced labor: Epidural analgesia and women’s perceptions of pain in early labor

**DOI:** 10.18332/ejm/99545

**Published:** 2018-11-16

**Authors:** Laura Kjær Jacobsen, Helle Haslund, Christina Brock, Birgitte Schantz Laursen

**Affiliations:** 1 Clinical Nursing Research Unit, Aalborg University Hospital, Aalborg, Denmark; 2 Clinic of Anesthesia, Children, Circuits and Women, Aalborg University Hospital, Aalborg, Denmark; 3 Mech-Sense (Center of Pain and Gatroenterology Research), Department of Gastroenterology and Hepatology, Aalborg University Hospital, Aalborg, Denmark; 4 Department of Clinical Medicine, Aalborg University, Aalborg, Denmark

**Keywords:** epidural analgesia, induction of labor, childbirth, labor pain, medically induced labor, spontaneous labor

## Abstract

**INTRODUCTION:**

Approximately 25% of all deliveries in Denmark are medically induced, typically characterized by more intense uterine contractions. The aim of this paper is to investigate the differences in the administration of epidural analgesia and pain experience between spontaneous and medically induced labor in nulliparous and multiparous women.

**METHODS:**

This is a prospective case-controlled study of 100 participating women in labor. The primary outcome was the timing of administration of epidural analgesia, by delivery progression and frequency. Pain scores were indicated by the McGill Pain Questionnaire and the duration of pain was also notified.

**RESULTS:**

In nulliparous and multiparous women, medically induced labor was associated with earlier administration of epidural analgesia in relation to the onset of labor pain, compared to women with a spontaneous onset of labor (10.4 vs 26.10 hours, p=0.0). There was a trend, however not statistical, in the use of epidural analgesia in relation to delivery progression, assessed as dilation of the cervix (3 cm vs 4.5 cm, p=0.07) and towards higher frequency for medically induced labor (51.5% vs 32.8%, p=0.07). In nulliparous women, a reduced period of labor pain was shown in medically induced deliveries compared to spontaneous deliveries (9.30 vs 19.00 hours, p=0.03). However, no significant differences in experienced pain were shown (Score: 28.70 vs 29.60, p=0.194).

**CONCLUSIONS:**

Epidural analgesia was administered earlier, and duration of experienced pain was shorter in medically induced labor, in comparison to spontaneous deliveries. However, the experienced pain was not different, possibly explained by a more intense labor process.

## INTRODUCTION

In Danish obstetric practice, labor is medically induced in approximately 25% of childbearing women. With 61397 births in 2017 within Denmark, approximately one in four were medically induced, which means that this intervention affects a large percentage of laboring women. However, no previous studies have been conducted on the effects of medically induced labor on women’s experiences of labor pain as a basis for this intervention.

In a review of qualitative research, Van der Gucht and Lewis^1^ identified a disparity between laboring women’s wishes to enhance their ability to cope with labor pain and the reality of clinical practice. To facilitate appropriate care of the individual woman in labor, Klomp et al.^2^ emphasized the need for midwives to recognize that women might prefer different approaches to the management of their labor pain. Wee^3^ found that the women who were most satisfied with their birthing experiences were those who felt satisfied with the analgesia and felt that they had received good pain relief, and he concluded that ‘*It is essential to provide timely information, support and choice of pain relief tailored to the individual parturient.*’. In a meta-analysis, Hodnett^4^ suggested that the involvement of the woman in the decision-making process during parturition has a positive effect on her level of satisfaction and her perception of labor pain.

Sparse research has been conducted specifically regarding the experience of labor pain in relation to the induction of labor. Nuutila et al.^5^ found that women who were induced reported a fear of pain less often than women who experienced the spontaneous onset of labor. In a qualitative analysis of postpartum interviews, Henderson and Redshaw^6^ concluded that labor pain was one of the main themes that emerged in relation to the induction of labor, and furthermore, they found that women who were induced were less satisfied with the care they received during labor. Accordingly, Hildingsson and Nystedt^7^ concluded that the induction of labor is associated with a less positive birth experience. Additionally, Heimstad et al.^8^ found that women who were induced experienced more frequent and more intense contractions. Petersen et al.^9^ studied the use of epidural analgesia in relation to the onset of labor and found that the induction of labor was related to an earlier administration of epidural analgesia. Furthermore, Hildingsson and Nystedt^7^ found that women who were induced received epidural analgesia more frequently. Nevertheless, no previous studies have explicitly examined the pain experience of women who were induced during labor to determine whether the induction has significant consequences for their overall pain experience. In Denmark, midwives offer primary support during parturition and often participate in the decision regarding what information will be provided to women prior to an induction of labor. Increased knowledge about women’s experiences of pain during medically induced labor could contribute to more informed midwifery support and the administration of pain relief during medically induced labor, and could enable the midwives to offer the parturient more accurate information prior to the induction.

We hypothesized that experienced pain is higher during medically induced deliveries compared to deliveries with a spontaneous onset of labor. Aim one was to characterize the demographics of nulliparous and multiparous women who were delivering after medically induced labor and after a spontaneous onset of labor. Aim two was to investigate the administration of epidural analgesia by analyzing the timing and the frequency of its administration. Aim three was to investigate the women’s experienced pain in terms of the perceived intensity and the duration.

## METHODS

### Design and participants

This study was a prospective case-controlled study at Aalborg University Hospital in Denmark. Data were collected from October 2014 until September 2015.

Inclusion criteria were nulliparous and multiparous women with no severe pathology related to their pregnancy, singleton pregnancy, vertex presentation, vaginal birth at term, fluent in Danish and expected to have either a spontaneous or a medically induced onset of labor. The medical induction of labor was accomplished using prostaglandin vaginal suppositories in the cases studied. The controls for this study were women who experienced a spontaneous onset of labor. Prior to inclusion, the women were informed both verbally and in writing by a midwife about the purpose of the study, which was done at a planned midwifery consultation that occurred at 36 weeks of gestation.

### Data collection

A midwife joined the participating women during their first encounters at the labor ward. The midwives attending the parturition were responsible for collecting the data. The midwives were instructed in the method that should be used for data collection and were supervised during data collection by the first author. There was no caseloading among the participants.

The administration of epidural analgesia was measured by the frequency in each group and in relation to the time of pain onset and the progression of the cervix. Data on the time of administration of epidural analgesia were analyzed in relation to the women’s perceptions of the onset of labor pain. This measure was chosen because an understanding of the women’s perception of duration of pain was important as this ensured the inclusion of women with long latent phases, which was central to the study’s objective. The McGill Pain Questionnaire was used to measure pain as it is a validated tool with a multidimensional conceptualization of experienced pain^10^. To ensure measurement validity, this study collected data of the pain experience during parturition as studies on the remembrance of labor pain have questioned the reliability of retrospective remembrance^11-13^.

The duration of labor pain was measured from the time when the women first experienced the beginning of continuous painful contractions leading up to the actual birth. The participating women indicated their experienced pain using the McGill Pain Questionnaire. Each woman scored her pain during the pauses between contractions, just before each vaginal exploration, for as long as she felt able to continue participating. Vaginal explorations were conducted at a 2- to 4-hour interval. For every completed McGill Pain Questionnaire, the time from the debut of labor pain, the dilation of the cervix and the mode of pain relief were noted. Options for pain relief were a birthing pool, acupuncture or a heating pad. If the women received epidural analgesia, the scoring of the pain ended. A background data questionnaire addressed the women’s gestational age, number of previous pregnancies, the onset of labor, the time of pain debut, the time of birth, the time of the epidural analgesia if administered, and the time of the cesarean section if it was necessary. If the data collection method was not strictly followed or if the data were inadequate, the case was disqualified, which occurred in seventeen cases.

### Statistical methods

The outcome-measures consisted of the administration of epidural analgesia measured in relation to the timing and frequency (primary endpoint of pain score), duration and a score of labor pain. Demographic characteristics of the women are presented as a mean with 95% CI, as a median and a range for continuous variables, and as frequencies for categorical variables. The study compared spontaneous versus medical onset of labor in nulliparous and multiparous women. Regarding the time-to-event variables, the period of labor pain is defined as the time from pain onset up until the time of labor; while the time to epidural analgesia is defined as the time from pain onset up until the administration of epidural analgesia. The median time-to-event for the variables above were estimated using the reverse Kaplan-Meier method and were presented as medians with 95% CI, and further compared using a logrank test. The score from the McGill Pain Questionnaire was a pain rating index, PRI (R), based on the result of a summative score between 0–78 and was calculated by the rank order of each word chosen by the women^10^. T-tests were used to compare the progression of labor alongside a comparison of the McGill Pain Questionnaire scores given by the spontaneous and medically induced labor groups and between nulliparous and multiparous women. Differences in categorical variables, such as epidural usage, smoking, or educational level between groups, labor start or parity, were tested using chi-squared tests for association. Analyses of the administration of epidural analgesia were conducted using Stata 13. Analyses of McGill Pain Questionnaire scores were conducted using Excel 2013. Results with a p-value below 0.05 were considered statistically significant. Some of the upper limits of the confidence intervals for the *median time-to-epidural analgesia/cervix at epidural analgesia* could not be calculated due to a lack of events/ epidural administrations after the estimated median.

### Ethical approval

No ethics approval was necessary according to The National Committee on Health Research Ethics, as the study was based on surveys and required no human biological material. The study was reported to the Danish Data Protection Agency (Journal no. 2008-58-0028). All of the participants freely gave their informed consent according to the Helsinki Declaration.

## RESULTS

### Aim 1: Demographics of participants

One hundred women were included in the study. There was no significant difference between the number of deliveries, the gestational age, the number of epidurals or the maternal ages, as can be seen in Table 1.

**Table 1:** Baseline characteristics of the cohort

*Baseline characteristics*	*N=100*	*Spontaneous onset*	*Medical induction*	*p*
Nullipara	67	42	25	
Multipara	33	25	8	NS
Gestational age days, mean (95% CI)	281.69 (280.01–283.37)	281.45 (279.80–283.10)	282.18 (278.32–286.04)	NS
Epidural anesthesia				
No	61	45	16	
Yes	39	22	17	NS
Maternal age years, mean ± SD	29.43 ± 4.36	29.56 ± 4.34	29.18 ± 4.45	
Nullipara	28.56 ± 4.06	28.64 ± 3.99	28.75 ± 4.02	
Multipara	31.31 ± 4.45	31.80 ± 4.11	30.76 ± 5.16	NS

### Aim 2.1: Time to administration of epidural analgesia

**Figure 1:**
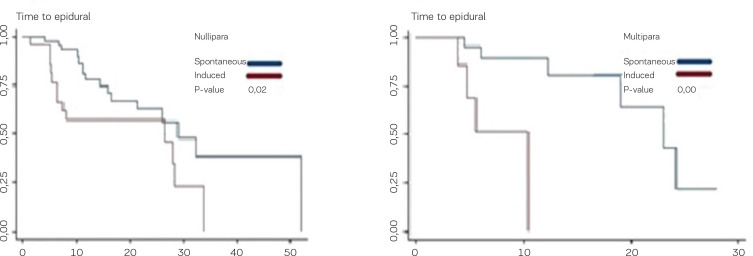
Timing of epidural analgesia. Time from the onset of labor pain up until the administration of epidural analgesia shown for women who were medically induced and for women who experienced spontaneous onset of labor. Left panel shows nulliparous women; right panel shows multiparous women.

In nulliparous women, the time in hours to the administration of epidural analgesia was shorter for medically induced deliveries (26.6 h; 95% CI: 6–40) compared with deliveries with spontaneous onset (29.0 h; 95% CI: 16–70), p=0.02. A similar outcome was found among multiparous women. Thus, the time for administration of epidural analgesia was shorter for medically induced deliveries among multiparous women (10.4 h; 95% CI: 3–90) compared with deliveries with spontaneous onset of labor (23 h; 95% CI: 12–30), p<0.00, which can be seen in Figure 1.

### Aim 2.2: Progression at administration of epidural analgesia

At the time of administration of epidural analgesia, the progression of the birth was assessed based on cervical dilation. In nulliparous women, the mean cervix was dilated 3.0 cm (95% CI: 2.00–4.00) in the group of 13 women who were induced and dilated 4.50 cm (95% CI: 3.5–5.5) in the group of 16 women who experienced spontaneous labor (p=0.33). In multiparous women, the mean cervix was dilated 4.0 cm (95% CI: 3.8–5.4) in the induced group of 4 women and dilated 3.5 cm (95% CI: 2.5–5.1) in the spontaneous group of 6 women (p=0.29).

### Aim 2.3: Frequency of administration of epidural analgesia

**Figure 2:**
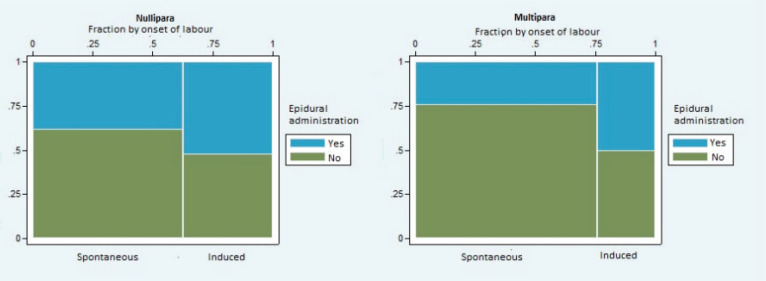
Frequency of epidural analgesia. Quantitative administration of epidural analgesia for women who were induced and for women who experienced the spontaneous onset of labor. Left panel shows nulliparous women; right panel shows multiparous women.

In nulliparous women, epidural analgesia was not administered at a significantly higher frequency in either the induced or the spontaneous labor groups (p=0.25). In multiparous women, epidural analgesia was administered at a significantly higher frequency in the induced group (p=0.04) compared to the spontaneous labor group, as seen in Figure 2.

### Aim 3.1: Qualitative experience of labor pain

When collecting data on the qualitative pain experience for nulliparous women, the progression of birth was assessed based on cervical status. In the induced deliveries the mean cervical status was a collum that measured 0.7 cm (SD ± 0.0) and a cervix that measured 1.6 cm (SD ± 1.1). In deliveries with a spontaneous onset, the mean cervical status was a collum of 0.3 cm (SD ± 0.5) and cervix of 2.5 cm (SD ± 1.0). Thus, women who were induced stopped reporting on the McGill Pain Questionnaire earlier with a mean 0.4 cm longer collum (p<0.00) and a cervix that was less dilated by a mean of 0.9 cm (p<0.00).

**Figure 3:**
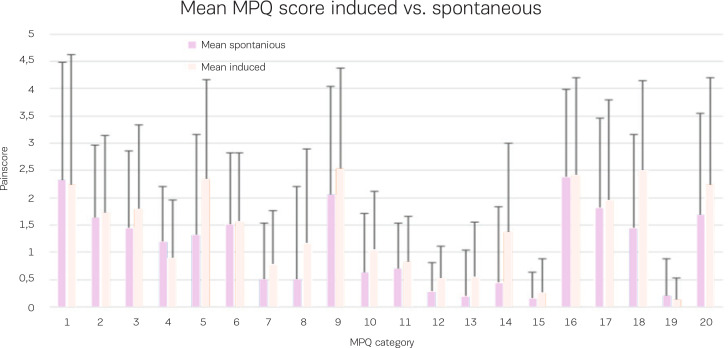
Mean pain score for nulliparous women who were induced and nulliparous women who experienced the spontaneous onset of labor based on the McGill Pain Questionnaire.

There was no association found between the mode of birth and the pain score or the women’s subjective assessment of pain. Comparing the experienced pain score between the induced deliveries and the spontaneous onset of labor deliveries, no significant difference was found in each of the McGill Pain Questionnaire categories or in the total summative average scores. The women who were induced scored their experienced pain at 29.6 points (95% CI: 24.7–34.5) versus 28.8 points in the spontaneous group (95% CI: 23.3–34.1), p=0.86, which can be seen in Figure 3.

### Aim 3.2: Subjective assessment of pain experience during labor

In nulliparous women who were medically induced, the duration of experienced labor pain was significantly shorter compared to the nulliparous women with a spontaneous onset of labor. On average, the nulliparous women who were induced gave birth 9.3 hours after the debut of pain (95% CI: 8.11–13.25) whereas the women with a spontaneous onset of labor gave birth 19.0 hours after the debut of pain, on average (95% CI: 15.28–22.32), p=0.03.

**Figure 4:**
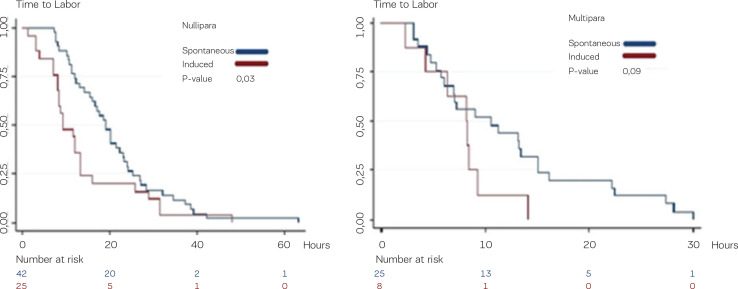
Duration of labor pain. The time from the onset of labor pain up until the birth for women who were medically induced and for women who experienced the spontaneous onset of labor. Left panel shows nulliparous women; right panel shows multiparous women.

In multiparous women who were medically induced, the duration of experienced labor pain was not significantly different from the multiparous women who experienced a spontaneous onset of labor. Women having a medically induced labor delivered 8.1 hours after the debut of pain (95% CI: 2.25–9.22) and women with a spontaneous onset of labor delivered 10.6 hours after the debut of pain (95% CI: 6.09–15.08), p=0.09, as shown in Figure 4.

## DISCUSSION

In nulliparous women, the time of the administration of epidural analgesia and the experienced pain duration was shorter in medically induced labor compared to spontaneous onset of labor. However, no difference was found in perception of labor pain or the frequency of administration of epidural analgesia. Finally, nulliparous women who were induced dropped out of the study significantly earlier in the labor process compared to nulliparous women with spontaneous onset of labor. In multiparous women who were induced, epidural analgesia was administered at a significantly higher frequency. However, we found no difference in the time of the administration of epidural analgesia or in the duration of experienced pain between multiparous women who were induced and those who had a spontaneous onset of labor.

The finding showed that nulliparous women who were induced typically received epidural analgesia earlier, which aligns with the findings of Petersen et al.^9^ that demonstrated a reduced interval from the onset of active labor to the administration of epidural analgesia. Medically induced labor in nulliparous women neither increased the experienced pain in early labor significantly, when compared to that of the women experiencing spontaneous onset of labor, nor did it influence the subjective characteristics of the experienced pain. However, the medically induced group received epidural analgesia earlier and declined to fill out the McGill Pain Questionnaire at an earlier stage in labor. Midwives conducting data collection reported that the dropout was due to inadequate resources to respond to the questionnaire between contractions. This may indicate that women who are medically induced perceive their overall pain in early labor with a greater intensity, which makes it more difficult for them to collaborate on the pain scoring exercise. Heimstad et al.^8^ found that the induction of labor was related to more frequent and more intense contractions. The fact that women who were induced received an epidural earlier and dropped out prior to the women with spontaneous onset of labor may support Heimstad et al.’s findings. In a qualitative study, Simonsen and Maimburg^14^ found that women experiencing postterm pregnancy and the induction of labor felt abnormal, felt as if they had failed, and felt stigmatized due to the pathologizing of their pregnancy and their birthing process. Furthermore, they felt distanced from the midwife due to a depersonalized approach in communication regarding post-term pregnancy and the induction of labor. This indicates that midwives may use a different approach to communicate with women who will be induced and that the emotional aspects involved may cause these women to have different needs. Hence, earlier and more frequent administration of epidural analgesia may also be related to a need for adjusted approaches for midwifery support and a different communication style to be used with women who are induced, compared to those who are experiencing spontaneous labor. Also, there may be differing requests from these two groups of women based on their mental perception of their situations.

Wee^3^ found that the most satisfied women were those who received good pain relief during labor. Consequently, due to our findings that the induction of labor is related to an earlier administration of epidural analgesia and a shorter duration of labor pain, a deduction could be made that the induction of labor causes a better birthing experience. However, this conclusion contrasts with the variation of 87 hours in experienced duration of labor pain among women who were induced. Furthermore, it compares unfavorably to the findings of Van der Gucht and Lewis^1^, Klomp et al.^2^, and Hodnett^4^, who emphasized the need for midwives to involve the women in the decision-making process and to recognize that they may need to use different approaches to pain management because some women want to be enhanced in their ability to cope with labor pain rather than receiving medical pain relief.

### Limitations

Data must be interpreted cautiously. First, it must be understood that the analysis of labor pain in this study is based upon of women’s pain experience from the debut of pain only up until early active labor. Secondly, the sample was non-randomized since in Danish obstetric practice, an induction of labor will only be carried out for medical reasons. Thirdly, the cohort of multiparous women only included 25 women who were induced and 8 women with a spontaneous onset of labor. Fourthly, a selection bias cannot be ruled out. Therefore, the data from this study may not be generalizable to accurately depict the birthing experience of all women.

## CONCLUSIONS

Taken together, these findings indicate that due to a more intense overall pain experience at an earlier stage in labor and a great variation in the experienced duration of labor pain, the induction of labor may give rise to unique care needs related to different approaches to emotional support and alternative pain relief options. Midwives must acknowledge the way in which the experiences of women who are induced might differ from those of women who experience a spontaneous onset of labor so that they can accommodate their individualized needs with additional support, analgesia, and an involvement that is individually tailored. To strive for better birthing experiences among both nulliparous and multiparous women, future studies would ideally focus on examinations of how care during an induced labor can be best individually tailored and how a medical induction of labor affects a woman’s overall satisfaction with her labor experience.
